# SNAVI: Desktop application for analysis and visualization of large-scale signaling networks

**DOI:** 10.1186/1752-0509-3-10

**Published:** 2009-01-20

**Authors:** Avi Ma'ayan, Sherry L Jenkins, Ryan L Webb, Seth I Berger, Sudarshan P Purushothaman, Noura S Abul-Husn, Jeremy M Posner, Tony Flores, Ravi Iyengar

**Affiliations:** 1 Department of Pharmacology and Systems Therapeutics, Mount Sinai School of Medicine, New York, NY 10029, USA; 2 Systems Biology Center New York, Mount Sinai School of Medicine, New York, NY 10029, USA

## Abstract

**Background:**

Studies of cellular signaling indicate that signal transduction pathways combine to form large networks of interactions. Viewing protein-protein and ligand-protein interactions as graphs (networks), where biomolecules are represented as nodes and their interactions are represented as links, is a promising approach for integrating experimental results from different sources to achieve a systematic understanding of the molecular mechanisms driving cell phenotype. The emergence of large-scale signaling networks provides an opportunity for topological statistical analysis while visualization of such networks represents a challenge.

**Results:**

SNAVI is Windows-based desktop application that implements standard network analysis methods to compute the clustering, connectivity distribution, and detection of network motifs, as well as provides means to visualize networks and network motifs. SNAVI is capable of generating linked web pages from network datasets loaded in text format. SNAVI can also create networks from lists of gene or protein names.

**Conclusion:**

SNAVI is a useful tool for analyzing, visualizing and sharing cell signaling data. SNAVI is open source free software. The installation may be downloaded from: . The source code can be accessed from:

## Background

Interactions between signaling pathways in mammalian cells indicate that a large-scale complex network of interactions is involved in determining and controlling cellular phenotype [1-3]. To visualize and analyze these complex networks, the biochemical networks may be abstracted to directed graphs [4]. To understand the topology of such networks, graph-theory methodologies can be applied to analyze networks' global and local structural properties [5]. Additionally, the value of assembled network datasets is enhanced with network visualization software and web-based information systems. These systems provide summary information, order, and logic for interpretation of sparse experimental results [6,7]. Visualization tools and web-based navigation systems provide an integrative resource that aids in understanding the system under investigation and may lead to the development of new hypotheses.

Graph-theory methods have been used in other scientific fields to analyze complex systems abstracted to networks. For example, Watts and Strogatz [8] defined a measure called the "clustering coefficient" (CC) for characterizing the level of clustered interactions within networks by measuring the abundance of triangles in networks (three interactions among three components). For instance, if a node has four neighbors and three of the neighbors are directly connected, the CC for that node is 0.5 because the four neighbors can be connected maximally with six links (3/6 = 0.5). The network's CC is the average CC computed for all nodes. Caldarelli *et al*. [9] formulated an algorithm to consider rectangles (four interacting nodes) in the clustering calculation, and called it the grid coefficient. Watts and Strogatz also used the characteristic path length to measure the disjointedness between nodes in networks. Characteristic path length is the average shortest path between any two pairs of nodes. It is calculated for all possible pairs of nodes, such that the average minimum number of steps between all pairs of nodes is the characteristic path length. Together, the CC and the characteristic path length measurements have a predictable relationship when computed for most real networks. This observation is called the "small-world" phenomenon [8].

Barabasi and coworkers [10] analyzed the connectivity distribution of metabolic networks and other biochemical networks and observed a connectivity distribution termed "scale-free". Scale-free property indicates that the connectivity distribution of nodes follows a long heavy tail that fits a power-law. Such distribution results in few highly connected nodes that serve as hubs whereas most other nodes have few links. Another topological property that is used to statistically analyze biochemical regulatory networks is the identification of network motifs. In biochemical regulatory networks, motifs are subcircuits of molecular interactions involving multiple cellular components. The different possibilities for subcircuit configurations made of several components define different types of network motifs. All the possible combinations for interconnectivity made of few components in directed graphs can be determined [11] and then used to identify their prevalence by comparing the counts in random topologies. This method was used to characterize motifs in gene regulatory networks from *Caenorhabditis elegans *and *Saccharomyces cerevisiae *[11-14]. This type of analysis identified signature patterns of network motifs that can characterize different types of networks, including signal-transduction networks [13,14]. The graph-theory based network analysis methods described above are statistical. Such statistical analysis of signaling networks requires that the size of the network is large enough (requiring an estimated minimum of 200 nodes). SNAVI includes functions to compute the clustering, characteristic path length, and connectivity distribution of networks, and provides the means to identify and visualize network motifs.

Statistical analysis of network topology is complemented by effective network visualization and web-based navigation tools. Maps or diagrams of signaling pathways help summarize many interactions at once. Maps may suggest new interpretations for experiments, because the act of preparing the maps imposes logical interpretation [15]. Additionally, mapping a network is an important initial step for developing models for quantitative simulation [16]. Molecular interaction network maps are constantly changing as new data become available, and manually redrawing signaling maps is not convenient or desirable. The requirements for mapping large-scale biological networks include showing an appropriate level of detail, minimizing overlap of nodes and links, and compatibility with multiple data storage formats [7].

Existing software tools draw networks automatically from databases, Excel spreadsheets, XML (Extensible Markup Language), or text files where interactions are listed in a structured format. One recommended platform is Cytoscape [17]. General network visualization software are often used by computational biologists, for example the Pajek software project [18], or AT&T's Graphviz project [19], where the second is an open-source project used as a library in many applications, i.e., Science Signaling uses GraphViz to display their Connections Maps [20]. When maps expand beyond a certain number of nodes (~40–50) it becomes impossible to follow the links generated using the Pajek (version 1.10) or GraphViz (version 1.0) programs. One solution is implementing zooming and panning functionality using scalable vector graphics (SVG) code [21] or Flash, or dividing large, complex pathways into sets of smaller interrelated pathways. Another solution is to allow users to specify a portion of the network they want to explore and then construct subnetworks that are easily navigated. SNAVI can be used to construct and visualize such subnetworks to allow investigation of larger networks.

SNAVI is a software tool for statistical analysis and visualization of large-scale cellular signaling networks and other biochemical intracellular networks. Here, we demonstrate how SNAVI can be used for web-based visualization and statistical analysis of biological regulatory networks. As an example, the installation of SNAVI provides a network representing signaling pathways in hippocampal neuronal cells [1]. To create this network, direct interactions were extracted from primary papers into a template stored in a flat file (Table 1), and then verified through a multistep manual review process by biologists. The network currently contains 594 nodes and 1422 links extracted from 1296 articles. Users may use this dataset or may load their own data. The process of creating, analyzing, and visualizing signaling networks using SNAVI is described in the methods.

**Table 1:** SNAVI native file storage format SIG file

Columns are separated by one white space and contain the following information:	
**Field**	**Description**

Source Name	Cellular component that is affecting a target component

Source Human Accession	Swiss-Prot accession code or other code if available

Source Mouse Accession	Swiss-Prot accession code or other code if available

Source Type	The type of molecule of this component

Source Location	Cellular localization of the component

Target Name	Cellular component that is affected by the source component

Target Human Accession	Swiss-Prot accession code or other code if available

Target Mouse Accession	Swiss-Prot accession code or other code if available

Target Type	The type of molecule of this component

Target Location	Cellular localization of the component

Effect	activation (+), inhibition (_), or neutral (0)

Type of Interaction	type of chemical interaction linking the two components

PubMed IDs	PubMed database accession number

## Implementation and results

### Overview and data files

**Figure 1:**
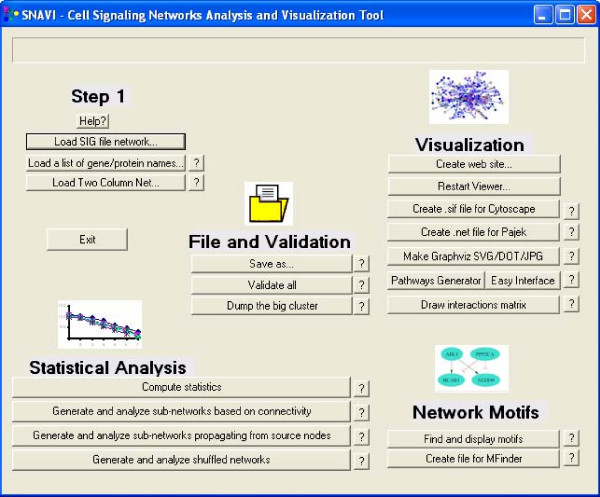
**The main SNAVI user interface**. The main menu is a simple dialog where the different functions are organized into a set of clickable buttons.

SNAVI is organized into seven functional parts: Loading data into SNAVI, file manipulation and validation, exporting data to other programs, network visualization, statistical analysis of network properties, and identification of network motifs. The main SNAVI interface divides the functions of the software into five sections (Figure 1). Help is available within the software by clicking the question mark buttons, which either opens a new window with detailed information or pops up a brief description of the function in question. SNAVI installation script automatically places a set of sample data files into the "Data" directory under the directory in which the SNAVI program was installed. These are "SNAVI.sig", "BACKGROUND.sig", "GENESLIST.txt", and "TWOCOL.txt". When analyzing user-supplied datasets, it is recommended that each dataset be located in its own directory.

The native data format for SNAVI conforms to a specified format (Table 1) and these files are called "sig" files. These are flat text files that contain lists of interactions where each interaction has 13 attributes. Table 1 also includes four interaction records given as examples, representing the 13 attributes from a "sig" file. There are three ways to load data into SNAVI for analysis: Users may load sample datasets that are included with the software ("sig" format), create a network from lists of human Entrez Gene official symbols using a background network within SNAVI that contain over 7,000 human protein-protein and ligand-protein interactions, or analyze user-supplied interaction data listed in two-column text format. Once loaded, datasets may be saved as "sig" format for later analysis or manual editing.

SNAVI contains several routines to validate the integrity and completeness of loaded network datasets. These routines check for name, type of nodes, type of interactions, and logical or syntactical inconsistencies in accession codes. For example, two different accession numbers associated with the same node name are flagged and reported, as well as interactions involving the same pairs of node-names but having different effects. All validation errors may be ignored, if the errors are not relevant to the user's analysis and visualization requirements. Most functions will still work even when validation errors exist. Validation errors are reported in a window created from a file called "validation.htm" that is automatically saved in the same directory or folder in which the loaded data file is from, which is referred to as the current working directory.

In most networks, not all nodes are reachable from all other nodes. This means that networks are fragmented into isolated but internally connected components. Some statistical measurements and visualization functions can only work for networks where all nodes are reachable from all other nodes. Therefore, SNAVI provides a mechanism to export the biggest component in a network into a new sig file for analysis. This file is created with the "Dump the biggest component" option on the File and Validation section of the main SNAVI interface.

#### Creating a network from a list of gene or protein names

Another acceptable input for SNAVI is a flat text file (".txt") containing a list of human genes or proteins. This type of file may be useful for creating an interaction network from experimental data, such as a list of mammalian genes or proteins produced by microarray or proteomic experiments. The gene or protein names must be listed in a text file and use the human Entrez Gene official symbol term for the corresponding human gene or protein in each row in the text file in a single column. SNAVI uses a background network of human protein-protein and ligand-protein interactions containing over 7,000 interactions to build a subnetwork containing the list of gene names connected through intermediate proteins or signaling components. The user specifies the number of intermediates (1 through 3) that can be used to connect any two genes in the list. Once SNAVI has created a subnetwork from the list of gene names, the resultant subnetwork can be saved as a "sig" file.

### Visualizing networks

SNAVI provides several options for viewing entire networks or network motifs. The viewing options involve html files, SVG files, jpg, and dot files depending on the visualization option selected. The color and shape settings for the graphical displays are stored in a small text file called colors_and_shapes.txt, which can be found in the current working directory. This file may be modified manually to generate user-defined colors and shapes. The file must have three columns in each row. The first column corresponds to the molecule type defined in the sig file, the second column is a color name defined in at  by GraphViz, and the third column is a shape defined at  by GraphViz.

After loading a network or creating a network from a list of gene names, one of the visualization options SNAVI provides is the creation of a set of web pages that are saved in the directory that contains the uploaded data file. When the program is creating the web site, it attempts to connect to PubMed and Swiss-Prot databases through an Internet connection to obtain information about the proteins and details about references used. The web page files include a main page that has links to pages containing the following information in addition to a network diagram organized by the type of component in the network: (i) an alphabetized list of molecules with links to individual pages for each node in the network, (ii) network statistics, (iii) legend to colors and shapes. The web pages created for each node contain an SVG map with all the upstream and downstream links from that node, and a list of references and detailed text descriptions of interactions directly implicated with each node. The pages also contain links to the Swiss-Prot database and statistics for individual nodes.

#### PathwayGenerator

**Figure 2:**
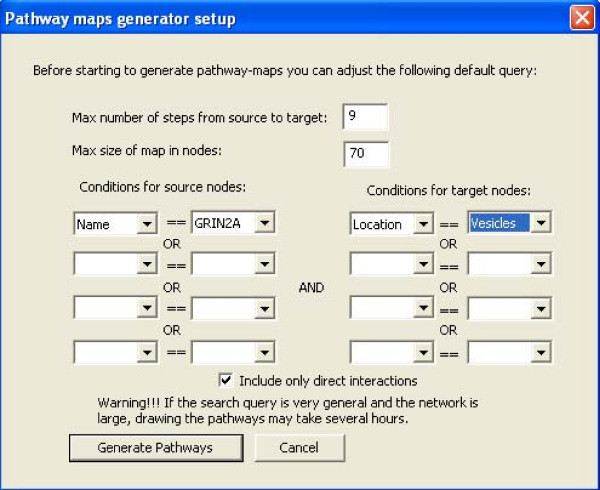
**PathwayGenerator example**. Pathway maps generator user interface dialog box.

**Figure 3:**
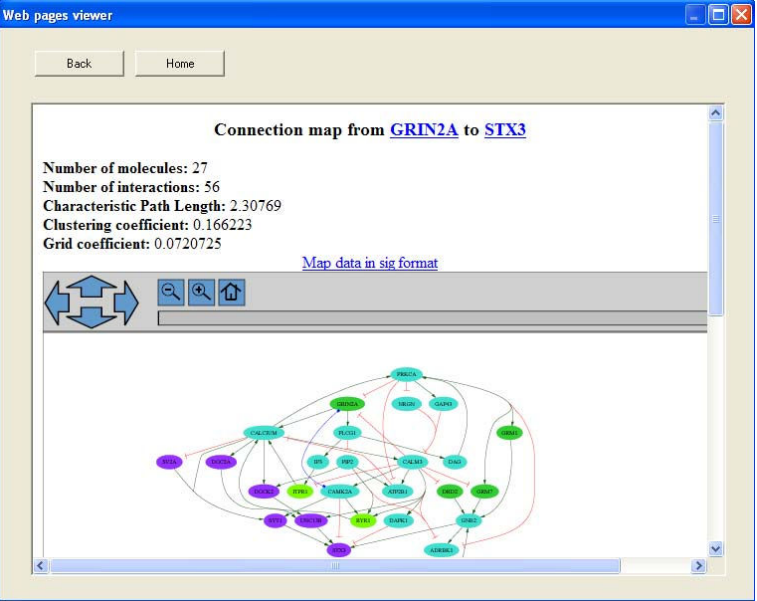
**PathwayGenerator example**. Pathway from GRIN2A to STX3 created automatically.

SNAVI's PathwayGenerator function provides a mechanism to query a network by allowing the user to select a source node and a target node and then finding the paths that connect these two elements in a network (for example, a ligand as source, and a downstream effector, such as a transcription factor, as target). The output is a graphical network map showing the interactions that connect the selected nodes. The program attempts to find the shortest paths from the source to the target while optimizing the maximum number of steps from the source to the target so that the map is of optimized size. This is performed to fit as many nodes and links as possible based on the user's specified parameters. The process of maximizing the number of steps from the source to the target is implemented to generate maps that are optimally sized for easiest navigation and maximal content, and to control execution time. An example of the interface and a generated map from GRIN2A to Syntaxin3 is provided (Figures 2 and Figure 3).

**Figure 4:**
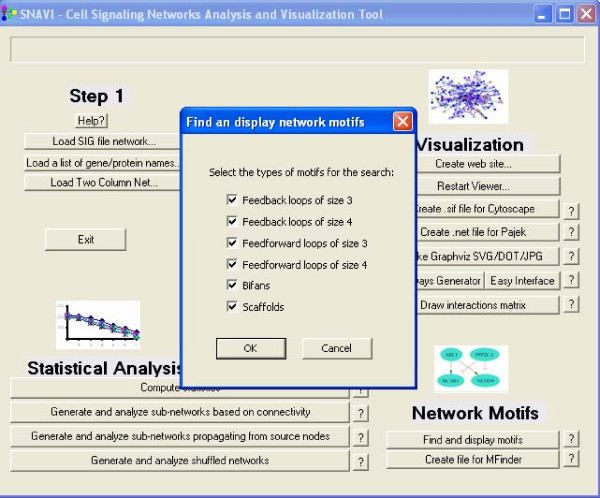
**Finding network motifs example**. Selection box for different types of network motifs.

**Figure 5:**
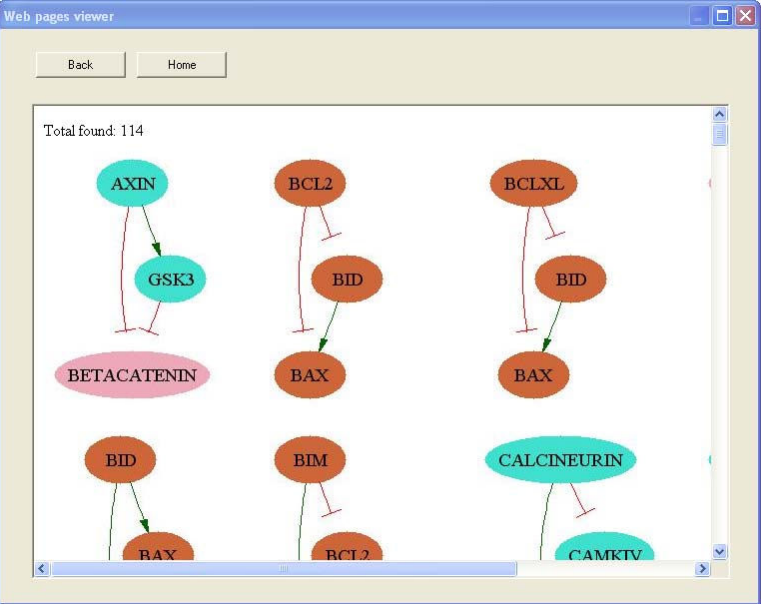
**Finding network motifs example**. Visualization of 3-node feed-forward network motifs.

There are two ways to create the query that draw subnetworks. The "PathwayGenerator" interface is the "advanced" user interface where the user may set the maximum number of parameters. The "Easy Interface" limits the options for the source node to those classified as ligands or receptors, and then allows the target node to be one of the following: transcription factor, kinase, phosphatase, vesicle-related protein, cytoskeleton, or ion channel. When the radio button next to each option is clicked a drop-down menu with the names of the nodes provides the available choices for each. SNAVI also includes a function that finds, counts, and plots a subset of small-sized network motifs: scaffolds (three-node triad of all neutral links), feedback loops, feed-forward loops and bifans [11]. An example of the user interface dialog box to select different motifs and a sample output of such analysis is provided (Figures 4 and Figure 5).

### Network statistics

The statistics report in SNAVI shows the number of components, interactions, and references for the network. The components are categorized according to their function and their locations. The interactions are categorized according their effects and the types of interaction. SNAVI statistics reports provide several standard graph-theory measures. For example, the clustering coefficient (CC) measures the level of clustered interactions in networks by measuring the abundance of triangles. The grid coefficient measures the level of clustered interactions by counting rectangles. The connectivity distribution is the number of nodes that share the same degree of connectivity.

To further investigate the topological structure of networks, SNAVI can be used to partition the loaded networks into subnetworks based on two different specified criteria. In the first option, which is accessed by the "Generate and analyze subnetworks based on connectivity", the inclusion of nodes and interactions is based on nodes' degree. In the second option, which is accessed by the "Generate and analyze subnetworks propagating from source nodes", the inclusion of nodes and interactions is based on specifying a group of nodes from which the connectivity is expanded in steps to build subnetworks. For example, subnetworks can be generated and analyzed by setting ligands as the source nodes and expanding the subnetworks in steps in the downstream direction. Starting from a specified group of source nodes, series of subnetworks can be created and analyzed expanding in steps from source nodes. The subnetworks characteristics produced by such analysis are reported in a HTML table. This table can be exported to an Excel spreadsheet.

Comparing real topologies to shuffled networks is important for identifying topological properties that have been selected for in biological regulatory networks. SNAVI provides the user with functions to create and analyze three types of shuffled networks based on the original loaded network: shuffled networks in which pairs of target nodes were repeatedly swapped, shuffled networks in which the connectivity is the same as that in the original network but the signs of effects (positive +, negative -, or neutral 0) are shuffled, or Erdos-Renyi networks [22]. Erdos-Renyi networks have the same number of nodes and links as the original network; however the links are randomly assigned between nodes.

### Exporting networks

Networks loaded into SNAVI can be exported into the following file formats: MFinder, Cytoscape, DOT and Pajek.

#### Creating a file for MFinder

The MFinder program [14] is a command line program that counts and reports network motifs. The program identifies statistically over- or under-represented network motifs, and reports the motifs that have been found in an output text file. The MFinder program accepts input as text files containing three columns of integers. The first two columns represent the source node and the target node, respectively. The third column is not used (reserved for future implementations).

#### Creating a file for Cytoscape

Cytoscape  is an open source bioinformatics software platform for visualizing molecular interaction networks and integrating these interactions with gene expression profiles and other state data. SIF files created with SNAVI can be loaded into Cytoscape for visualization. This file can be loaded into Cytoscape using the File->open function.

#### Creating a file for Pajek

SNAVI users can also export networks into the Pajek software . Pajek is Windows-based freely available network visualization and analysis software. Pajek accepts formatted text files as input that follow the Pajek language specification. Files are saved with the .net extension. The Pajek files created with SNAVI specify the node colors based on their degree of connectivity (k), as well as by assigning colors to links (green for activation arrows, and red for inhibition arrows) and specifying the direction of the links so that arrows are drawn. The Pajek software is mostly useful for drawing large networks. It contains algorithms that optimize locations of nodes to minimize link lengths and link crossings.

### Comparing SNAVI to Cytoscape

Many of the functionalities that have been implemented for SNAVI are available with the network analysis software system Cytoscape. Although the SNAVI and Cytoscape software systems provide some similar functionality, Cytoscape is much more robust and provides many more features than SNAVI. Hence, SNAVI is not as stable as Cytoscape and as such we do not recommend SNAVI as the first choice for biological network analysis in general. However, SNAVI still has some unique features that are not found in Cytoscape. SNAVI is especially appropriate for analysis and visualization of mammalian cell signaling networks represented as directed graphs. This is because SNAVI's core functions were developed specifically for the analysis applied for a study published in Science in 2005 [1]. A case study of how to use SNAVI to analyze and visualize this signaling network is provided as supporting material to this article [see additional file 1]. The two main features in SNAVI that are not available in Cytoscpae are: a) several specific open source algorithms for performing different types of complex network analyses; b) SNAVI can effectively be used to created complete web-sites from large-sized networks by breaking a large network into pieces presented as linkable web-pages. We summarized the differences and similarities between SNAVI and Cytoscape in Table 2 as per the peer reviewers' request. Although many features are shared by SNAVI and Cytoscape users should consider that although the same features might be implemented there could be still significant differences in the way such features were implemented. Hence, there are advantages/disadvantages and/or user personal preferences for specific implementations.

**Table 2:** Feature comparison between SNAVI and Cytoscape

**Feature**	**SNAVI**	**Cytoscape**
• Users can dynamically change the location of nodes	**SNAVI**: Not available, users have the option to export the network to Pajek or Cytoscape	**Cytoscape**: Users can move the nodes on the screen

• Different options for network layout	**SNAVI**: Fixed visualization using presetting of parameters from GraphViz	**Cytoscape**: Several rendering algorithms for dynamic organization of nodes on the screen

• Coloring of nodes	**SNAVI**: Can be specified in a text file	**Cytoscape**: Can be specified dynamically using built-in feature in the software

• Zooming and panning	**SNAVI**: Offered with some SVG output maps	**Cytoscape**: Embedded within the main framework

• Computing network statistics	**SNAVI**: One functions that counts node types, and computes connectivity distribution, characteristic path length, clustering and grid coefficients	**Cytoscape**: Many network measures for nodes links, and stats for the entire network can be computed with the NetworkAnalyzer plug-in

• Finding and displaying network motifs	**SNAVI**: Available for only specific types of motifs size 3–5 nodes	**Cytoscape**: Available using the Metabolica, Network Motif Finder, and NetMatch plug-ins

• Finding paths from source to target	**SNAVI**: Available with the PathwayGenerator algorithm	**Cytoscape**: Available with the ShortestPath and PeSca plug-ins

• Generating random networks	**SNAVI**: Different types of random networks: Erdos-Renyi, Barabasi-Albert, Random Swaps can be created from loaded real networks	**Cytoscape**: Random Network Plug-in was announced as a Google Summer of Code project

• Linking to background knowledge of protein interactions	**SNAVI**: Limited to one function that uses a background human protein interaction network	**Cytoscape**: Can be linked to many different background networks using the BioNetBuilder, BioPAX, MiMI and cPath plug-ins

• Computing network parameters as connectivity propagates through the network	Only in SNAVI implemented specifically for Ma'ayan et al. (1)	

• Creating web-sites from networks in text file	Only in SNAVI implemented specifically for Ma'ayan et al. (1)	

• Connecting a list of genes using a background network	**SNAVI**: Limited to one function that uses a background human protein interaction network	**Cytoscape**: Can be done with several of the plug-ins

• Network clustering using the MCODE plug-in;	Not in SNAVI	Only in Cytoscape

• Linking to microarray data;		

• Ability to generate filters; Linking to Gene Ontology with the binGO and GOlorize plug-ins; Linking to domain-domain putative interactions with the DomainNetworkBuilder plug-in; Linking to protein structure; Linking with text mining tools; Network inference algorithms; Ability to merge networks		

## Conclusion

Effective analysis and visualization tools for large-scale cellular networks that can draw connectivity diagrams with an unbiased predisposition from large interaction datasets can help cell and molecular biologists identify potential new pathways and sometimes predict missing components and interactions. Such tools can be used to support hypothesis generation and experimental design, improve presentations in seminars and publications, and serve as a valuable educational resource for students. The abstraction of signaling networks into nodes linked through three types of links (activating, inhibiting, or neutral) is simplified, but nevertheless captures key features of information flow in cellular regulatory networks. Others have proposed more detailed descriptive and visualization approaches, which may provide more information about cellular regulatory systems than what is currently provided by SNAVI [15,23]. Although these other visualization schemes can be useful, it is more difficult to annotate interactions to fit into their templates. In order to move from gene and protein lists to network maps and then to predictive models of mammalian cells, signaling networks will indeed have to become more detailed in their annotation. Such details will include kinetic parameters and spatial information [24]. We hope that SNAVI will provide one more resource towards achieving such long term goals.

## Availability and requirements

Project name: SNAVI

Project home page: 

Operating system: Windows XP or Vista

Programming language: Visual C++ .NET version 7.1

Other requirements: Adobe SVG Viewer Version 3.03, WinGraphViz.

License: GNU GPL

Any restrictions to use by non-academics: License is not needed but interested commertial parties should contact technology@mssm.edu before reusing the code.

## Authors' contributions

AM designed the system and implemented the code except for the grid coefficient and matrix visualization functions which were implemented by SPP. RLW, SIB, NAH, JMP, TF tested the system. AM and SLJ wrote the manuscript. RI reviewed the manuscript and supervised the project.

## Supplementary Material

Additional File 1 **Case Study Instructions for SNAVI.** The document provides step by step instructions on how to use SNAVI to analyze and visualize a large cell signaling networks provided with the software. Click here for file
