# Supplementary material for: SNAVI: Desktop application for analysis and visualization of large-scale signaling networks
Source: BMC Syst Biol. 2009 Jan 20;3:10. doi: 10.1186/1752-0509-3-10 (PMC2637233; doi:10.1186/1752-0509-3-10)
Supplement: Additional File 1 — Case Study Instructions for SNAVI. The document provides step by step instructions on how to use SNAVI to analyze and visualize a large cell signaling networks provided with the software. [file 1752-0509-3-10-S1.pdf]

Case Study Instructions for SNAVI

Version 2.5 January 2009

Spplemental Material for the software article:

SNAVI: Desktop Application for Analysis and Visualization of Large-Scale Signaling Networks

Avi Ma'ayan, Sherry L. Jenkins, Ryan L. Webb, Seth I. Berger, Sudarshan P. Purushothaman, Noura Abul-Husn, Jeremy M. Posner, Tony Flores, and Ravi Iyengar

Department of Pharmacology and Systems Therapeutics, Systems Biology Center New York, Mount Sinai School of Medicine, New York, NY 10029, USA

Installing SNAVI

1. Download the SNAVI_setup.zip file.

Note: You might need to download the SVG viewer http://www.adobe.com/svg/ if the computer does not already have this plug in.

2. Extract the zip file. The zip file contains one file: SNAVI_setup.exe.
3. Double-click the SNAVI_setup.exe file to install the software.
4. Read the terms of use and complete the installation.

Note: If a previous version of SNAVI is already on your computer, you will need to uninstall it (using "Add or Remove Programs") before installing a newer version.

Loading Data

1. Click the "Load SIG file network…" button under Step 1.
2. Locate and select the appropriate SIG file in the C:\Program Files\SNAVI\Data folder, for example the "SNAVI.sig" file.
3. Click the "Open" button.
4. After receiving the following message, "The network was loaded successfully!" click the "OK" button.
5. Click the "Validate all" button under File and Validation and review the report.

Note: This step is optional. The SNAVI.sig file contains errors, which are listed in the validation report. However, the file may still be used for analysis.

6. Click the Back button to return to the main SNAVI interface and proceed with analysis, visualization, or export.
7. If desired, correct any errors by opening the sig file in a text editor, editing the file, and saving.
8. If desired, export the largest set of connected nodes using the "Dump the big component" button. If the file was edited or the biggest component is preferred, reload the updated or new sig file using the "Load SIG file network… button".

Creating a network from a list of gene or protein names

1. Click the “Load a list of gene/protein names…” button under Step 1.
2. Select the appropriate "txt" file, for example GENELIST.txt, and click the "Open" button.
3. Select the maximum number of intermediates (1-5) from the drop down box in the next window and then click the “OK” button.

Note: Selecting more than one intermediate will considerably slow down the execution time. The GENELIST file has 23 genes and takes approximately 30 minutes with the number of intermediates set to 1.

4. After receiving the following message, “A subnetwork containing your genes/proteins was successfully created!” click the “OK” button.
5. Click the "Save as…" button to save the file in sig format.

Note: The resulting file may have some missing data (listed as NA) and some interactions listed as neutral (0).

6. Click the "Validate all" button and review the report.

Note: If desired, open the sig file in a text editor and modify the data to eliminate inconsistencies or errors, or use the "Dump the big component" option to export the connected set of nodes, then reload the updated or new sig file using the "Load SIG file network…" button.

Loading two-column networks

1. Click the “Load Two Column Net…” button under Step 1.
2. Select the appropriate file, for example "TWOCOL.txt".
3. Click the "Save as…" button under File and Validation to create a "sig" file.
4. Manually edit the sig file using a text editor and save.
5. Reload the updated sig file using the "Load SIG file network…" button.
6. Click the "Validate all" button and review the report.

Note: If desired, open the sig file in a text editor and modify the data to eliminate inconsistencies or errors, or use the "Dump the big cluster" option to export the connected set of nodes, then reload the updated or new sig file using the "Load SIG file network…" button.

Visualizing Networks

1. Load the SNAVI.sig file.

Note: Datasets in any of the acceptable file formats may be loaded using the appropriate button in Step 1 on the main SNAVI interface.

2. Press the “Create web site…” button under Visualization to initiate the web site creation process.

Note: This may take several minutes depending on the size of the network.

If the viewer does not automatically open a window to the main web page of the network, press the “Restart viewer” button.

3. Click any of the symbols representing the classes of components to view a list of those components including the type and location of the component, the number of

links (relations) to that component, the CC, and the average path length. For the SNAVI.sig example, click the symbol called "Membrane".

Note: An alphabetized list of components in the network may also be accessed by selecting the "Index of molecules" from the main page.

4. Click any of the components in the list to view of the upstream and downstream components connected to the selected component and the annotation regarding each interaction displayed. For the SNAVI.sig example, click the GRIN2A component in the list.

Note: If the component is highly connected, the viewer will include a set of SVG tools to zoom in; zoom out; pan left, right, up and down; and restore the image to the original setting (the house symbol).

6. To return to the previous page of information, use the "Back" button. To return to the main index page, use the "Home" button.

7. To run the web pages through a web browser instead of through SNAVI, go to the subdirectory containing the data file, open the directory that includes "_website as part of the name, and open the file called index.htm.

Note: ActiveX controls or scripts must be enabled in the web browser.

PathwayGenerator

1. Load the SNAVI.sig file.

Note: Data sets in any of the acceptable file formats may be loaded using the appropriate button in Step 1 on the main SNAVI interface.

1. Click the "Pathways Generator" button to open the "Pathway maps generator setup" dialog box.
2. Select specific nodes or groups of nodes as source components (the starting points of the network maps), and select the target, the downstream nodes, using the scrollbars. For this SNAVI.sig example, choose Name=GRIN2A for the source node and Location=Vesicles for the target node.

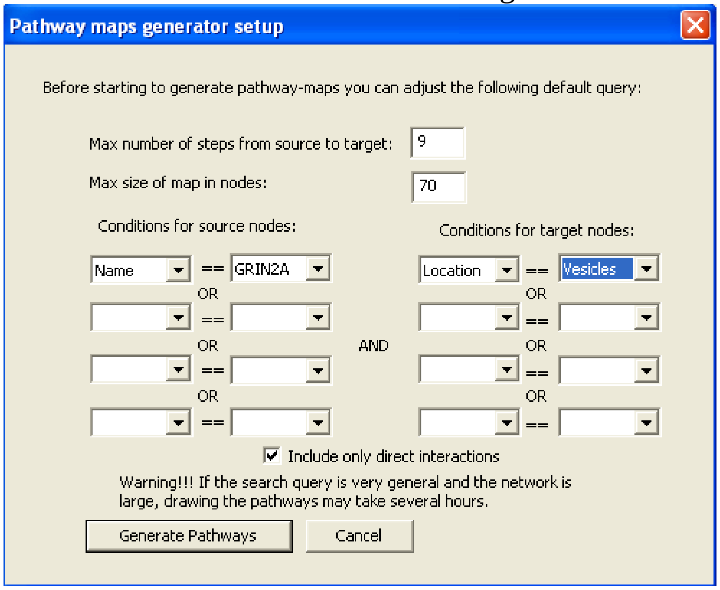

Note: The type, name, or localization of the node may be selected from the left side menu for each source and target and then the appropriate value is selected from the right side menu for each source and target.

3. Specify the maximum size of the network maps (number of nodes) and the maximum number of steps from source nodes to target nodes. For this SNAVI.sig example, the default maximum number of steps and maximum size of the map were used.
4. Click the “Generate Pathways” button.

Note: The sample query took approximately 15 minutes.

5. If no pathways exist between the two source and target nodes, then the system will return a message stating "No pathways with the following query were found".
6. If one or more paths exist connecting the source and target nodes, then choose a single source and single target component from the list and click the "Draw Pathways" button. For this SNAVI.sig example, choose GRIN2A as the source component and STX3 as the target component.

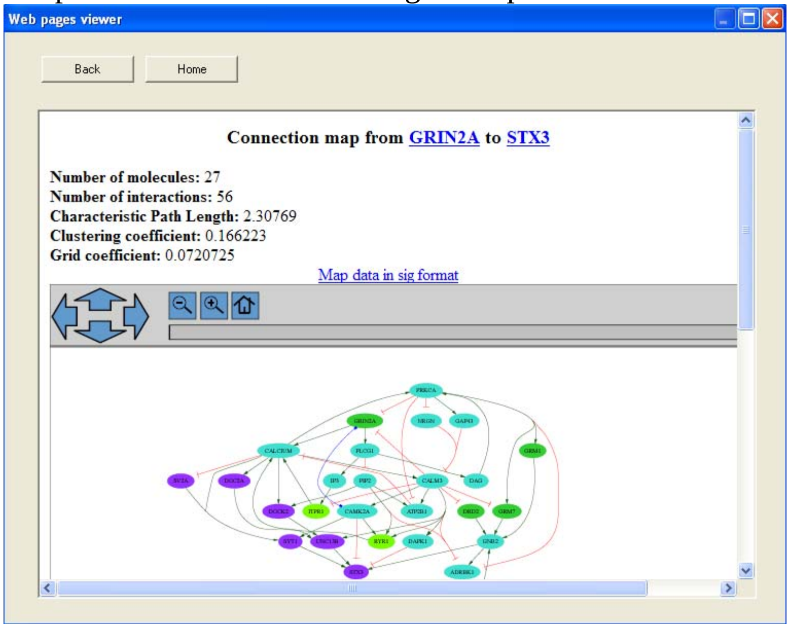

Note: SNAVI saves a set of files for the generated pathway. These are found in the “pg” directory and include an svg, jpg, and htm files named for the source and target nodes, and a colors_and_shapes.txt file, which dictates the symbols in the SVG view of the network map.

7. Click any of the node symbols to access information about the relationship.
8. Click "Map data sig format" to obtain a window containing the data, which can be copied and pasted into a text file to save as a sig for later analysis if desired.
9. Click the "Back" button to choose to draw a network map between a different source and target from the available list.
10. Press the "Home" button from the network map or the "Back" button from the source and target choice interface to return to the main SNAVI interface.
11. Click "Restart Viewer" to go back to the source and target choice interface.

Pathways Generator: Easy Interface

1. Load the SNAVI.sig file.

Note: Data sets in any of the acceptable file formats may be loaded using the appropriate button in Step 1 on the main SNAVI interface; however, the data must include the attributes for the type of molecule in order to use the Easy Interface.

2. Click the “Easy Interface” button to open the “Pathway Generator Easy Interface” dialog box.

3. Click the radio button next to one of the two source node options and then choose from the list of available nodes. (For example, select EGFR for the receptor source).

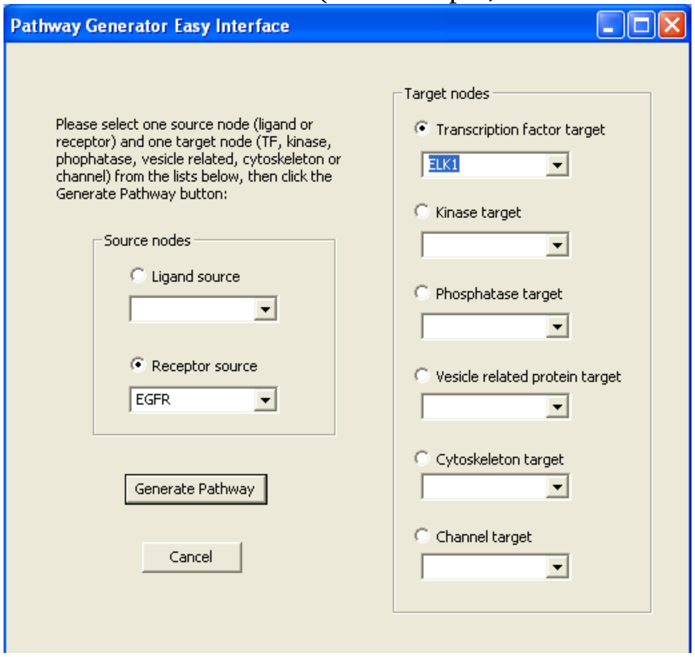

Note: Be sure that the radio button is checked.

4. Click the radio button next to one of the target node options and then choose from the list of available nodes. (For example, select ELK1 for the kinase target.)

Note: Be sure that the radio button is checked.

5. Click the "Generate Pathway" button.

6. Select the source and target node in the next window and click the "Draw Pathway" button.

Note: SNAVI saves a set of files for the generated pathway. These are found in the pg directory and include an svg, jpg, and htm file named for the source and target nodes, and a colors_and_shapes.txt file, which dictates the symbols in the SVG view of the network map.

7. Click each node to get additional details.

8. Click "Map data sig format" to obtain a window containing the data, which can be copied and pasted into a text file to save as a sig for later analysis if desired.

9. Click the "Back" button to choose to draw a network map between a different source and target from the available list.

10. Click the "Back" button from the source and target choice interface to return to the main SNAVI interface.

11. Click "Restart Viewer" to go back to the source and target choice interface.

Making Graphviz files

1. Load the TWOCOL.txt file.

Note: Data sets in any of the acceptable file formats may be loaded using the appropriate button in Step 1 on the main SNAVI interface; however, the data set must be fewer than 100 nodes.

2. Click the “Make Graphviz SVG/DOT/JPG” button, which will save three files in the working directory. If prompted, save the "sig" file.

Note: The filenames will be the same as the original data file and have the extensions ".dot", ".svg", and ".jpg".

3. View the hierarchical pathway image that opens in a SNAVI window or open the files with an SVG-enabled Web browser (svg file), or a GraphViz visualization tool (dot file), or any image software or Web browser (jpg file).

4. Click the "Back" button to return to the main SNAVI interface.
5. Click the "Restart Viewer" button to return to the hierarchical GraphiViz map.

Network Statistics

1. Load the SNAVI.sig file.

Note: Data sets in any of the acceptable file formats may be loaded using the appropriate button in Step 1 on the main SNAVI interface.

2. Click the "Compute Statistics" button.
3. Review the information or copy and paste the information into a text file for later use.

Note: Use the scroll bar to see the information at the bottom of the report.

Drawing an interactions matrix

1. Click the “Draw interactions matrix” button.
2. The “Interactions Matrix” window is displayed.

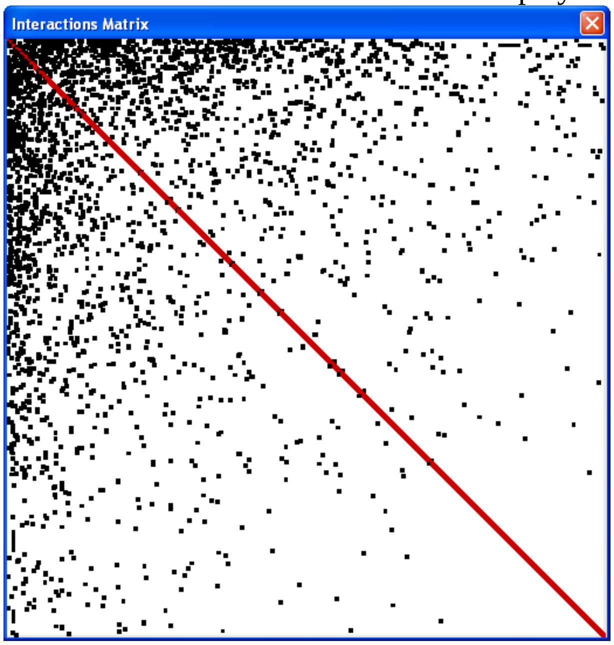

3. Pressing the PrintScreen button on the keyboard saves the image to the clipboard. Paste the contents of the clipboard into graphics drawing tools and crop the interactions matrix image.

Finding and displaying motifs

1. Click the “Find and display motifs” button.
2. A dialog box opens in which the types of motifs to be searched can be selected.
3. Press the “OK” button.

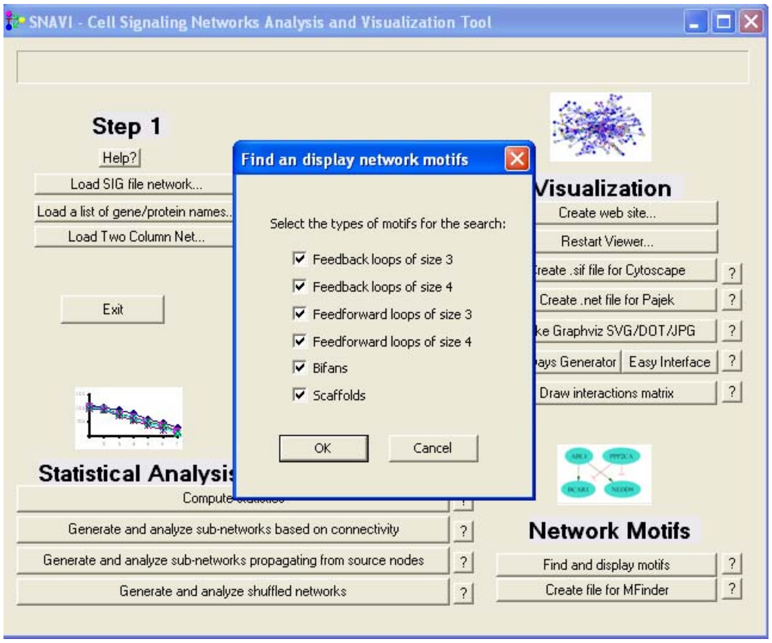

4. SNAVI finds the motifs, creates small-sized images, and displays an interface in a web-page in which all the motifs found can be viewed. All images and web-pages are stored in the current working directory.

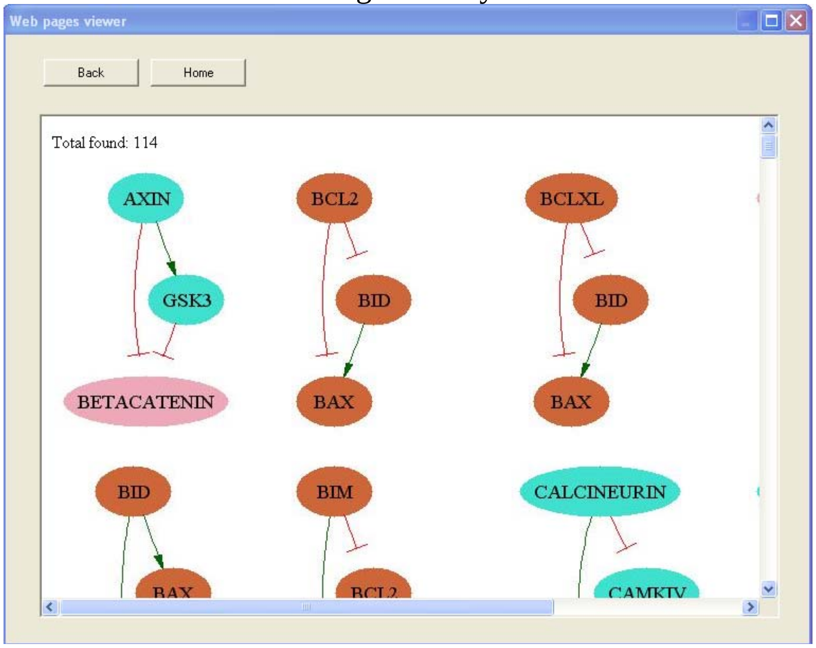

Exporting Files for Use in Other Programs

Creating a file for MFinder

1. Click the “Create file for MFinder” button.
2. SNAVI creates a file that can be used as input for MFinder (the default file is input_for_mfinder.txt).
3. The SNAVI output file can be analyzed with MFinder by typing the following from a DOS shell prompt: mfinder.exe "SNAVI output to MFinder.txt" -s "motifs size" -r "number of randomized networks to analyze" -f "output file with the reported results".
4. See the http://www.weizmann.ac.il/mcb/UriAlon/NetworkMotifsSW/mfinder/MfinderManual.pdf for more details

Creating a file for Cytoscape

1. Click the “create .sif for Cytoscape” button.
2. SNAVI creates a file called “input4cytoscape.sif” and saves it in the current working directory.
3. In Cytoscape version 2.5.2, the file created in SNAVI can be opened using the File>Open function.

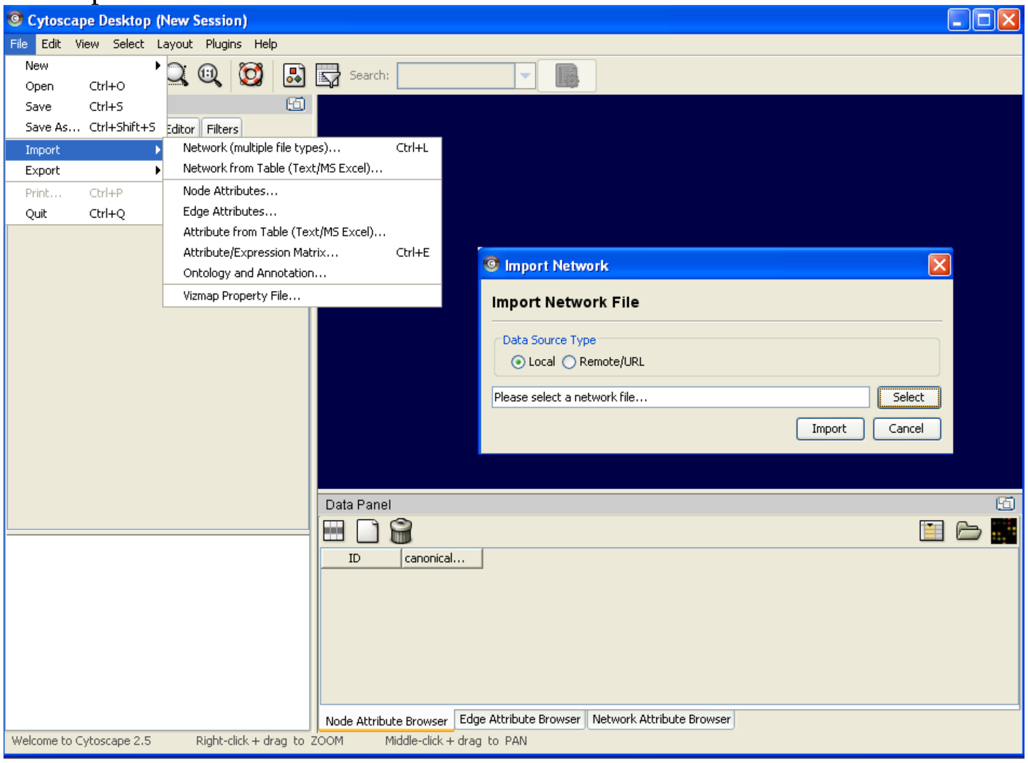

Creating a file for Pajek

1. Click the “Create a .net file for Pajek button”.
2. SNAVI creates a .net file in the current working directory.
3. Open Pajek and load the .net file.

4. Select the Draw function. The network will be drawn in a circular pattern.
5. Use the Layout->Energy->Kamada-Kawai->Free function for an alternative visualization.

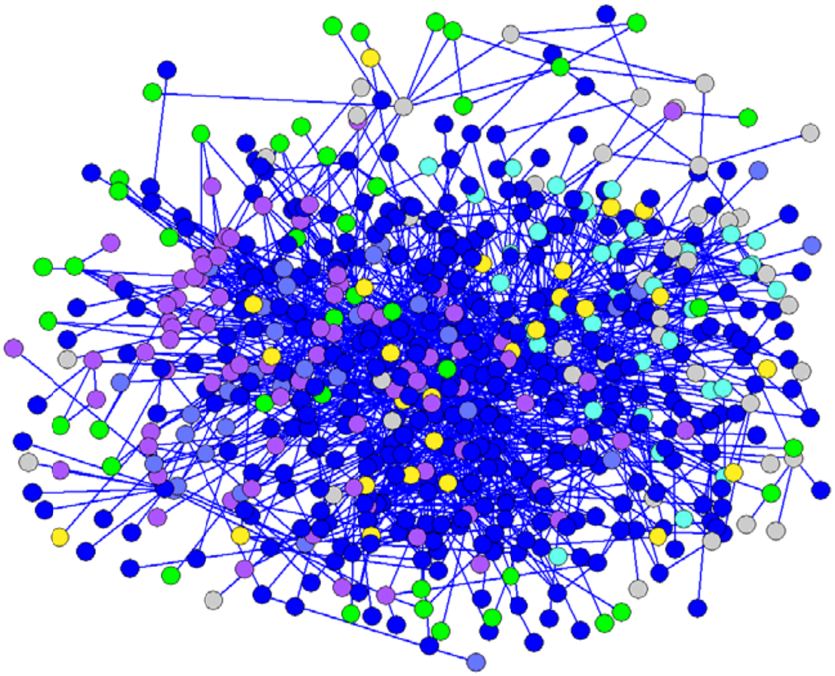

Contact and Additional Help

Any questions and/or suggestions should be send to avi.maayan@mssm.edu
